# Automated N-Glycosylation Sequencing Of Biopharmaceuticals By Capillary Electrophoresis

**DOI:** 10.1038/s41598-017-11493-6

**Published:** 2017-09-15

**Authors:** Marton Szigeti, Andras Guttman

**Affiliations:** 1 Horváth Csaba Memorial Institute for Bioanalytical Research, MMKK, University of Debrecen, Debrecen, Nagyerdei krt 98, 4032 Hungary; 2 MTA-PE Translational Glycomics Research Group, Research Institute of Biomolecular and Chemical Engineering, University of Pannonia, Veszprém, Egyetem u. 10, 8200 Hungary; 3 SCIEX, 250 S. Kraemer Blvd, Brea, CA 92821, USA

## Abstract

Comprehensive analysis of the N-linked carbohydrates of glycoproteins is gaining high recent interest in both the biopharmaceutical and biomedical fields. In addition to high resolution glycosylation profiling, sugar residue and linkage specific enzymes are also routinely used for exoglycosidase digestion based carbohydrate sequencing. This latter one, albeit introduced decades ago, still mostly practiced by following tedious and time consuming manual processes. In this paper we introduce an automated carbohydrate sequencing approach using the appropriate exoglycosidase enzymes in conjunction with the utilization of some of the features of a capillary electrophoresis (CE) instrument to speed up the process. The enzymatic reactions were accomplished within the temperature controlled sample storage compartment of a capillary electrophoresis unit and the separation capillary was also utilized for accurate delivery of the exoglycosidase enzymes. CE analysis was conducted after each digestion step obtaining in this way the sequence information of N-glycans in 60 and 128 minutes using the semi- and the fully-automated methods, respectively.

## Introduction

The rapidly increasing usage of glycoproteins as biopharmaceutical products has created a demand for fast, efficient and reliable bioanalytical techniques for comprehensive glycosylation analysis, including carbohydrate sequencing^1^. One of the fastest growing groups of these new generation protein therapeutics are monoclonal antibodies (mAbs) and fusion proteins containing the Fc portion of mAbs. MAbs, in most instances, possess a conserved N-linked glycosylation site on each of the C_H_2 domains of the Fc portion of the heavy chain of the molecule, but may also possess additional attached sugar structures at the Fab domains^2^. Increasing evidence shows that the carbohydrate moieties of biotherapeutics play important roles in their biological activity, physicochemical properties, and effector functions^3^. Even minor changes in these carbohydrate structures (linkage, position and site occupancy) can influence the effectivity of the products. The extremely high diversity of glycosylation makes structural elucidation of these complex carbohydrate molecules very challenging and in most instances only the combination of various methods can provide the desired information^4^. The most frequently used analytical methods for the structural elucidation of complex carbohydrates include capillary electrophoresis (CE), nuclear magnetic resonance spectroscopy (NMR), mass spectrometry (MS) and liquid chromatography (LC), often combined with with each other and/or with exoglycosidase digestion techniques^5^.

Attempts to sequence complex glycan molecules started in the late eighties in the last century by Khorlin *et al*., who used a variety of exoglycosidase enzymes and high performance liquid chromatography (HPLC) to analyze the structures of fluorophore labeled acid desialylated fetuin^6^. A detailed method was published later on microscale sequencing of N-linked carbohydrates released by hydrazinolysis and separated by chromatography^7^. Lectins in conjunction with exoglycosidases were also utilized for structural analysis of carbohydrates^8^. Characterization of protein glycosylation by exoglycosidase digestion and laser desorption mass spectrometry was described by several groups in the mid nineties^9–11^. At the same time, the first capillary electrophoresis based oligosaccharide sequencing was reported by Guttman^12^.

For the analysis of biological samples, Rudd *et al*. reported the release and analysis of sub-picomole levels of N-glycans directly from SDS PAGE gel bands utilizing MS and HPLC^13, 14^. Mechref and Novotny used mass spectrometric mapping to sequence N-linked carbohydrates from submicrogram amounts of glycoproteins^15^, while others applied ESI time-of-flight MS^16, 17^. Callewaert and coworkers used a multicapillary DNA sequencing instrument for large scale glycome mapping^18^. A two-dimensional array was applied by Tzur *et al*. for simultaneous sequencing of N- and O-linked carbohydrates using various lectins immobilized to a MALDI plate in the first dimension and sequential exoglycosidase digestion in the second dimension^19^.

Reusch *et al*. compared seven non-MS methods aiming Fc-glycosylation analysis and glycan structure identification of mAbs using multiple CE and HPLC platforms with various separation and labeling approaches^20^. A similar comparative study was organized by Lauc and coworkers aiming MS based methods for high throughput glycosylation analysis^21^. Based on different separation techniques and approaches on glycosylation analysis, the MIRAGE (minimum information required for a glycomics experiment) initiative was founded to establish guidelines for qualitative and quantitative results obtained by diverse types of glycomics analyses^22^. In addition, critical quality attributes (CQA) were established to ensure high quality and efficient results on Fc-linked glycan analysis and structural assignment^23^.

An early attempt towards automation was reported by Holland’s group, suggesting online glycan sequencing by phosphylipid assisted capillary electrophoresis^24, 25^. Packer and coworkers published a detailed protocol on structural analysis of N- and O-glycans using various techniques^26^. A recent report showed separation of N-linked carbohydrates from eight commercial recombinant mAb drugs by porous graphitized carbon (PGC) chromatography on a microchip in conjunction with electrospray ionization hybrid quadrupole time-of-flight (ESI-Q-TOF) MS that allowed to establish a glycan library of over 70 carbohydrate structures^27^. Special software toolsets were also developed for targeted use of exoglycosidase digestions in structural analysis of carbohydrates^28, 29^.

In this paper, we report on a novel automated approach for N-glycan sequencing of biopharmaceuticals using instrumental capillary electrophoresis. The sample compartment of the CE unit was used as a temperature controlled reaction chamber for the enzyme digestion steps accommodating both the semi- and fully-automated approaches. The separation capillary played a dual role as in addition to the CE analysis of the analyte molecules it was also utilized to deliver the appropriate enzymes into the reaction mixture in case of the fully automated setup.

## Results

In this study, rapid and automated exoglycosidase digestion based carbohydrate sequencing of human immunoglobulin G (IgG) and Enbrel (etanercept) fusion protein N-glycans was performed by utilizing the sample storage compartment of the CE instrument for reaction temperature control and the separation capillary as enzyme delivery device. Accurate temperature setting of the compartment allowed rapid exoglycosidase digestion reactions performed in the required temperature range of 40 °C to 60 °C. Commonly used approaches for glycan structure identification included both serial or array based exoglycosidase digestion of complex glycan pools by sequentially cutting off the individual sugar residues from the non-reducing end of the carbohydrate structures (in our case including sialic acid, galactose and N-acetylglucosamine residues). The resulting reaction products were identified by the CE migration time shifts of the affected peaks after each digestion step.

Please note that in our work the otherwise frequently used core fucosylation digestion was not included to avoid the highly structure specific results caused by the possible steric hindrance of the enzyme that might occur during the cleaving reaction. As a matter of fact, core fucosylated and non-core fucosylated structures can be readily distinguished by their CE migration time differences and the corresponding glucose unit (GU) unit values. Thus, core fucosylation removal was apparently not necessary for N-glycan sequence analysis of the biopharmaceuticals examined (IgG and Etanercept). Nonetheless, non-core fucosylation – if present –, as well as high mannose structures can be readily identified using GU value calculation from the glycan profile and search in the relevant databases (NIBRT – glycobase.nibrt.ie, HLBS – www.hlbs.org). The resulted electropherograms were aligned using co-injected bracketing standards (DP2 and DP15) and the GU values of the peaks were calculated after each sequencing step using our earlier published triple internal standard approach^30^. The actual sugar residue losses were identified from the IgG and Enbrel glycans by their GU shifts corresponding to the monosaccharides released as shown in Table 1. Their positional and anomeric information were specified by the exoglycosidases used. The shift values were considered to calculate and build up the glycan structures in the sample after all enzymes applied and the reaction products were analyzed by CE^4^.

**Table 1:** Identified migration time and GU values of IgG and Enbrel N-glycan pools at the corresponding separation temperatures.

Peak	Structure	Migration time (min)	GU value	Monoisotopic Mass [Da] NIBRT
**Human IgG glycan pool (Fig**. 2 **; 25 °C)**				
1	FA2G2S2	3.224	5.00	2368.84
2	FA2BG2S2	3.253	5.14	2571.92
3	FA2(3)G1S1	3.451	6.04	1915.69
4	FA2G2S1	3.605	6.80	2077.75
5	FA2BG2S1	3.643	6.98	2280.83
6	FA2	3.781	7.69	1462.54
7	FA2B	3.885	8.23	1665.62
8	FA2(6)G1	3.990	8.76	1624.60
9	FA2(3)G1	4.057	9.11	1624.60
10	FA2B(6)G1	4.070	9.18	1827.68
11	FA2G2	4.262	10.16	1868.70
12	FA2BG2	4.336	10.55	1989.73
13	F(6)M3	3.567	6.75	1056.39
**Enbrel glycan pool (Fig**. 3 **; 30 °C)**				
1	A2G2S2	2.767	5.18	2222.78
2	FA2G2S2	2.825	5.50	2368.84
3	FA2(6)G1S1	2.899	5.90	1915.69
4	FA2(3)G1S1	2.991	6.42	1915.69
5	A2G2S1	3.030	6.65	1931.69
6	A2	3.053	6.78	1316.49
7	M5	3.072	6.89	1234.43
8	FA2G2S1	3.119	7.17	2077.75
9	FA2	3.214	7.75	1462.54
10	FA2(6)G1	3.390	8.82	1624.60
11	FA2(3)G1	3.446	9.16	1624.60
12	A2G2	3.464	9.27	1640.59
13	FA2G2	3.619	10.22	1868.70
14	M3	2.737	4.94	910.33
15	F(6)M3	2.873	5.67	1056.39

N-linked glycans were first enzymatically removed from the IgG molecules by peptide N glycanase F (PNGase F) digestion, then labeled with aminopyrene trisulfonate (APTS), purified with magnetic beads and eluted with DDI water prior to CE-LIF analysis, as published earlier by Váradi *et al*.^31^. Capillary electrophoresis profiling was done before and after each sequencing reaction steps. The exoglycosidase digestion reactions (sequencing) were applied by using the appropriate enzymes to remove the corresponding individual sugar residues from the glycan pool for structural identification. Albeit, pH and temperature optimums of the various exoglycosidase enzymes may slightly vary, in our case the glycans were kept in water after our earlier described magnetic bead based purification process^31^, and optimized the reaction temperature without additional buffering steps (i.e., buffer exchange or addition). Sequencing reaction temperatures were controlled in the sample storage compartment of the capillary electrophoresis instrument up to 60 °C. A wireless thermometer was used to record the actual reaction temperature during the study. Despite of the possible conductive heat transfer through the air in the compartment, efficient temperature control was achieved even at 60 °C. A minimal temperature drop of up to 4 °C was observed during the injection process when the compartment door was open. However, the target temperature was reached again within a few minutes.

Please note that exoglycosidase digestion reactions are conventionally performed overnight at 37 °C, requiring one day for array based methods and several days in the serial enzyme reaction approach to complete the entire carbohydrate sequencing process. Utilizing the sample compartment to set the enzymatic digestion temperature in the reaction vials offered the following advantages for our CE based automated glycan sequencing approach: (i) The actual digestion and separation temperatures were decoupled.; (ii) Since the sequencing reactions were not conducted within the separation capillary the entire effective column length was available for separation also offering the user full control over separation parameter adjustments. (iii) The separation method was not limited to the use of special buffer systems and additional reaction plugs (different pH, salt and concentration) for on-column digestion. (iv) Provided the opportunity to follow the efficiency of the enzymatic reaction steps with *in situ* measurement of the samples during the processes, by simply injecting a small portion of the reaction mixture into the separation capillary; and finally (v) The developed method was simple, robust and completely system independent, thus, easily applicable for routine use. It is important to note that besides capillary electrophoresis, other liquid phase separation techniques (e.g., LC) could be also used provided accurate temperature control in the sample storage compartment in the given temperature range and the option of precise exoglycosidase enzyme delivery.

### Effect of temperature

**Figure 1:**
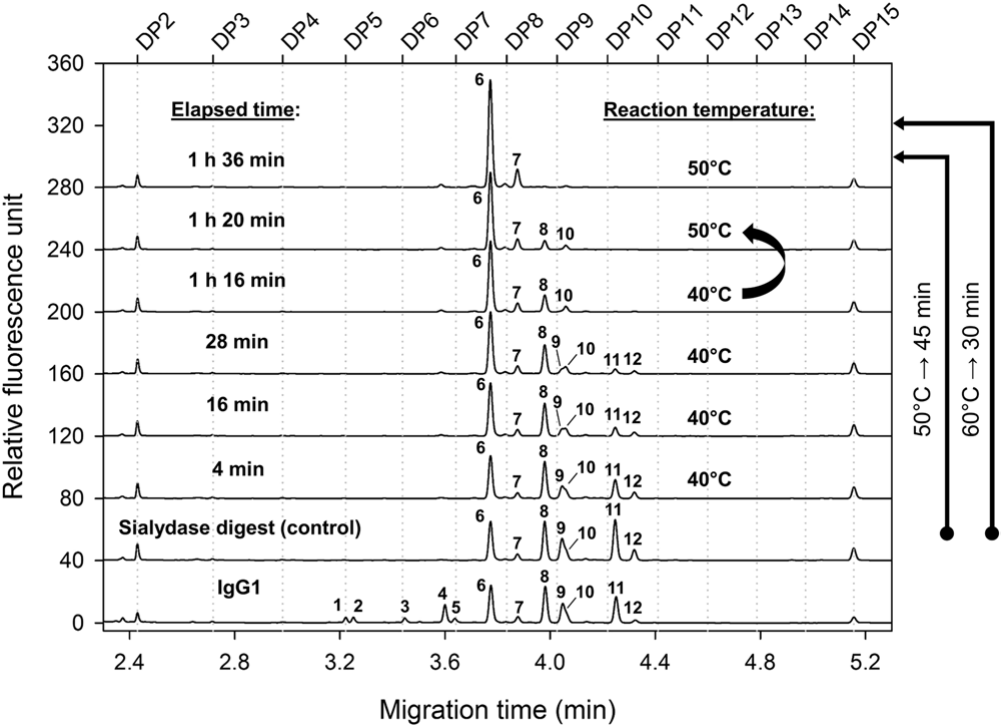
Reaction time and temperature optimization of the β-galactosidase digestion step on desialylated IgG N-glycans. Elapsed reaction times and the corresponding reaction temperatures are shown above the corresponding traces. Separation conditions: 20 cm effective length (30 cm total length, 50 µm ID) bare fused silica capillary; LIF detection (ex: 488 nm/em: 520 nm); 25 °C separation temperature; 30 kV (0.17 min ramp time) separation voltage in reversed polarity mode (1000 V/cm); Injection: 3.0 psi/5.0 min water −>3 kV/3.0 sec sample −>1.0 kV/1.0 sec bracketing standard.

The bottom trace in Fig. 1 shows the CE-LIF separation of the APTS labeled IgG N-glycan pool. The upper electropherograms compare the digestion performance of the β-galactosidase enzyme on the desialylated samples (control trace) at different temperatures using the incubation times (elapsed time) shown on the traces. First, the sample compartment temperature was set at 40 °C. At this temperature the β-galactosidase enzyme exhibited high cleavage capacity towards the FA2(3)G1, FA2B(6)G1 and FA2G2 structures (peaks 9, 10 and 11, respectively)(traces with 4, 16, 28 and 1 h 16 min elapsed times). However, the fact that peaks corresponding to FA2(6)G1 (peak 8) and FA2BG2 (peak 12) were still detectable even after 76 minutes, suggested that the digestion process for these structures was not complete at 40 °C. Thus, the reaction temperature was increased to 50 °C at the elapsed time point of 1 h 20 min that apparently accommodated the completion of the β-galactosidase digestion process for all structures within the remaining 16 minutes (1 h 36 min total elapsed time). The arrows in the right side of Fig. 1 depict other possible reaction routes showing that conducting the entire β-galactosidase digestion step at 50 °C or 60 °C, complete β-galactose removal were achieved in 45 or 30 minutes, respectively. Similar reaction temperature optimization was accomplished for the other two enzymes (sialidase and hexosaminidase) applied in this study to determine the shortest time required for complete removal of the corresponding sugar residues.

### Semi-automated glycan sequencing

**Figure 2:**
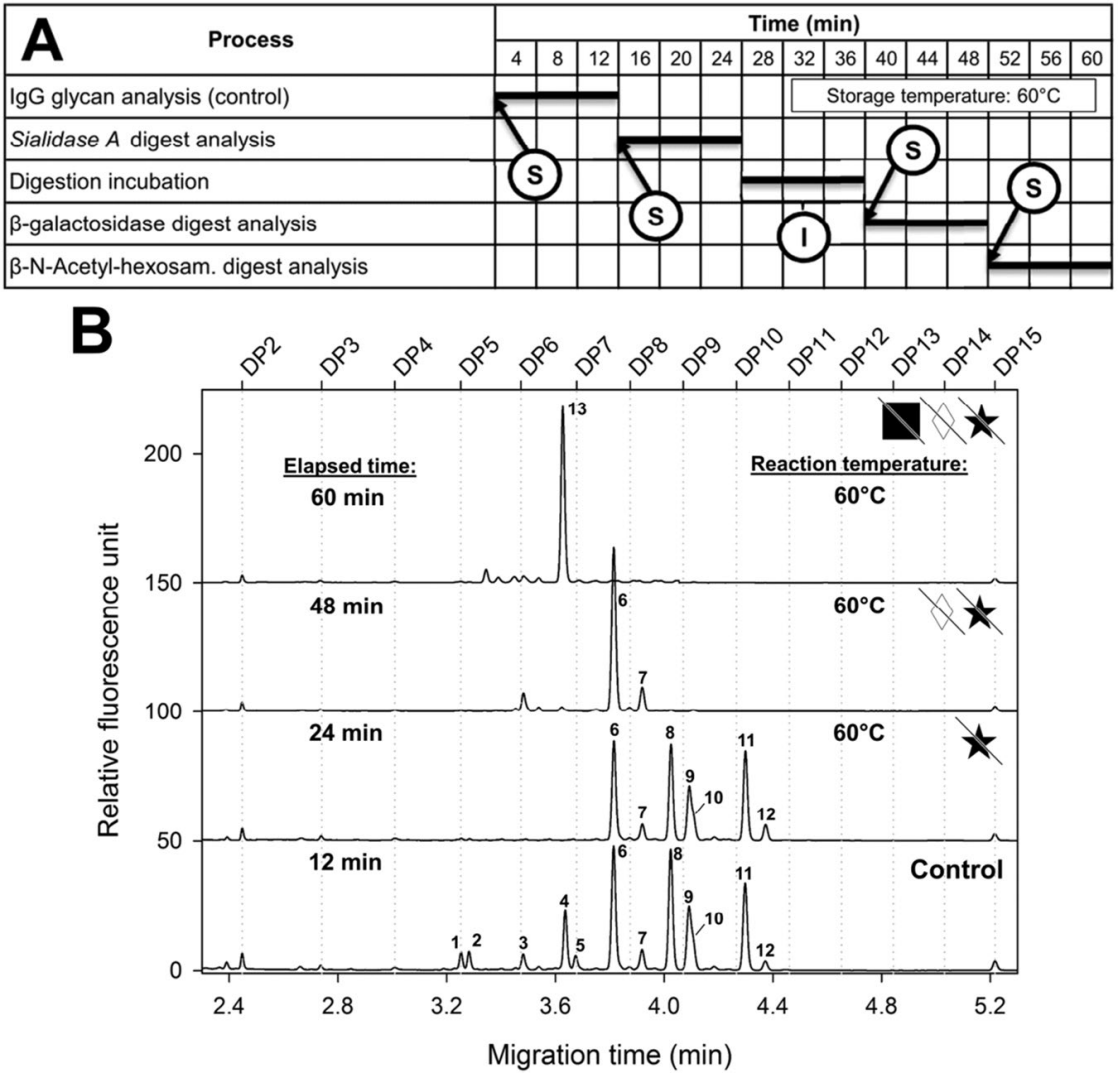
Gantt chart of the semi-automated method (Panel A) and the resulting electropherograms (Panel B) of the exoglycosidase array based digestion approach for IgG N-glycan sequencing. Ⓢ Time point of sample injection; Ⓘ﻿ incubation time. Separation conditions were the same as in Fig. 1.

Two techniques were developed utilizing the temperature controlled sample storage compartment as incubation chamber for automated exoglycosidase digestion based oligosaccharide sequencing. The semi-automated approach (Fig. 2, Panel A) required three parallel reaction mixtures with the relevant array of enzymes, i.e., sialidase, sialidase + galactosidase and sialidase + galactosidase + hexosaminidase. The released and APTS labeled IgG N-glycan pool was used as control (Fig. 2, Panel B, bottom trace). The corresponding reagent mixtures (1.0 µL volume each) containing 5.0 mU Sialidase A, 5.0 mU Sialidase A + 25 mU β-galactosidase and 5.0 mU Sialidase A + 25 mU β-galactosidase + 25 mM β-N-Acetyl-hexosaminidase, respectively, were added manually to the reaction tubes prior to the incubation step. The control and the three reaction mixtures were placed into the sample tray and incubated in the storage compartment at 60 °C. First, the undigested control sample was analyzed (12 min separation time) followed by the exoglycosidase enzyme array containing reaction mixtures after 12 minute (sialidase), 36 minute (sialidase + β-galactosidase) and 48 minute (sialidase + β-galactosidase + β-N-Acetyl-hexosaminidase) digestion times as depicted in Fig. 2, Panel A. Additional incubation time was applied after sialidase digestion to ensure full galactosidase and hexosaminidase release. The semi-automated approach required 60 min total glycan sequencing time including the CE-LIF separations. The resulting traces are shown in Fig. 2, Panel B.

### Fully automated glycan sequencing

**Figure 3:**
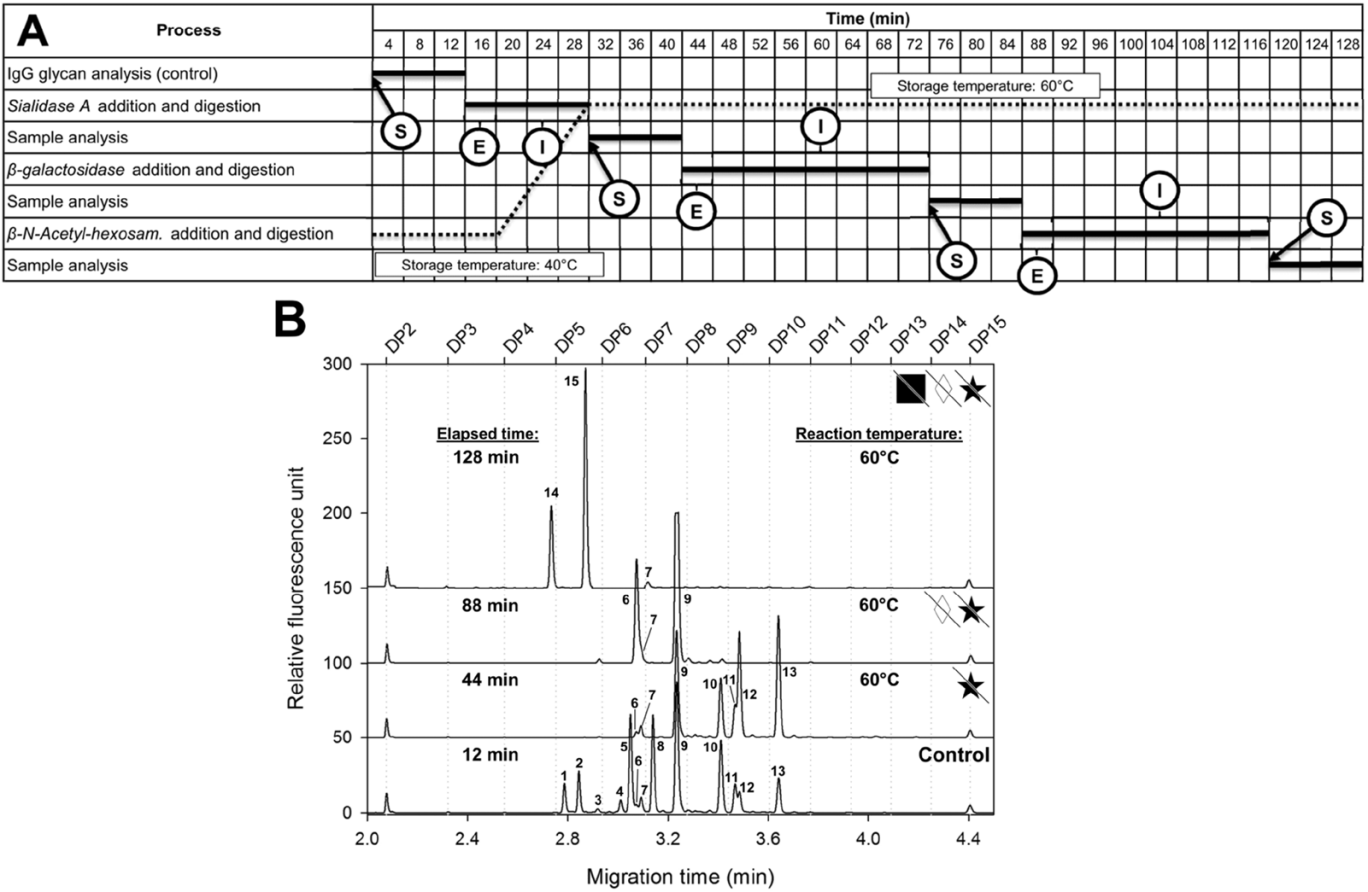
Gantt chart of the fully automated method (Panel A) and the resulting electropherograms (Panel B) of the consecutive exoglycosidase digestion steps based sequencing of APTS labeled Enbrel N-glycans. Ⓢ Time point of sample injection, Ⓘ incubation time, and Ⓔ time points of exoglycosidase enzyme addition. The dotted line depicts the temperature profile of the sample compartment during the exoglycosidase digestion process. Separation conditions were the same as in Fig. 1, except temperature was set to 30 °C.

The fully automated approach shown in Fig. 3, Panel A, on the other hand, required only one reaction vial, but consecutive capillary mediated delivery of the individual exoglycosidases. Small amounts of enzymes (5.0 µL) were placed in the nano vials in the outlet buffer trays of the capillary electrophoresis unit. After the initial CE-LIF analysis of the APTS labeled N-glycan pool of Enbrel (Control trace in Fig. 3, panel B), the separation capillary was used as an injection device to deliver the corresponding enzymes required for the consecutive digestion reactions (1.0 µL from each that were achieved by using 80 psi for 0.5 min in a 30 cm total length capillary with 50 µm ID). The incubation times used in this instance were 12, 28 and 28 minutes after Sialidase A, β-galactosidase and β-N-Acetyl-hexosaminidase addition. The reaction mixture was automatically injected and analyzed by CE-LIF after each incubation step. During this fully automated process, the sample compartment temperature was first set to 40 °C for the sialidase treatment. After 16 minutes, the temperature was increased to 60 °C using a 12 minute ramp time to accommodate the reactions of the other two enzymes and kept at that temperature until the end of the entire sequencing process (dotted line in Fig. 3A). This fully automated approach required 128 min total processing time because of the consecutive digestions steps. Figure 3, panel B shows the individual sequencing separation traces of the APTS labeled Enbrel glycan pool during the fully automated process injected at 28, 72 and 116 minutes from the reaction vial, prior to the pressure mediated delivery of the next exoglycosidases trough the separation capillary in reverse direction mode to initialize the individual sugar residue release reactions. Please note that in this instance the 30 °C separation temperature resulted in better resolution for the Enbrel glycans^32^, so all GU values and shifts were calculated accordingly (Table 1).

## Discussion

This is the first paper describing, rapid semi- and fully-automated exoglycosidase digestion based carbohydrate sequencing utilizing the temperature control feature of the sample storage compartment of a capillary electrophoresis instrument. The actual temperatures of the reaction mixtures were closely monitored during the sequencing process using a wireless thermometer. By optimizing the reaction parameters (temperature, enzyme concentration, incubation time), complete digestion of the individual sugar residues and CE analysis of the resulting product profiles were achieved in 60 minutes using the semi-automated and in a little over 2 hours by the fully automated glycan sequencing approaches. In contrast, using conventional methods to accomplish this task may take up to four days. Furthermore, the actual digestion and separation temperatures were decoupled resulting in no requirement of special buffer systems and processing the enzymatic reactions within the separation capillary, accommodating high-resolution glycan analysis.

## Methods

### Chemicals and Reagents

Water (HPLC grade), acetonitrile (HPLC grade), 1 M NaBH_3_CN (in THF) and human immunoglobulin G (IgG) were from Sigma Aldrich (St. Louis, MO, USA). Enbrel (etanercept) was kindly provided by the Semmelweis University, Budapest, Hungary. Peptide N-glycosidase F (PNGase F) enzyme (200 mU) and the specific exoglycosidases of Sialidase A (α(2 → 3,6,8,9)R; ), β-galactosidase (Jack Bean; β(1 → 3,4,6)R; ) and β-N-Acetyl-hexosaminidase (Jack Bean; β(1 → 2,3,4,6)R; ) were from ProZyme (Hayward, CA, USA).

### Capillary Electrophoresis

Capillary electrophoresis measurements with laser induced fluorescence detection (CE-LIF) were performed on a PA800 Plus Pharmaceutical Analysis System (SCIEX, Brea, CA). Separations were accomplished in a 50 µm ID, 20 cm effective length (30 cm total length) bare fused silica (BFS) capillary. All CE separations were performed either at 25 °C or 30 °C by applying 30 kV (0.17 min ramp time) in reversed polarity mode (cathode at the injection side, anode at the detection side) resulting in 1000 V/cm electric field strength. A three stage sample injection was applied to accommodate field amplified sample introduction: (1) water at 5.0 psi for 5.0 sec; (2) sample at 3.0 kV for 3.0 sec; and (3) bracketing standard (DP2 and DP15) at 1.0 kV for 1.0 sec. Data acquisition and analysis was done using 32Karat software package (version 10.1, SCIEX).

### Sample preparation and exoglycosidase digestion

Immunoglobulin G1 (100 µg) and Enbrel (etanercept) (100 µg) were used for the experiments employing the Fast Glycan Labeling and Analysis Kit (SCIEX) for protein denaturation, enzymatic glycan release (using PNGase F, 2.5 mU), fluorophore labeling (8-aminopyrene-1,3,6-trisulfonate, APTS) and excess dye removal by magnetic beads. The sequencing (exoglycosidase digestion) reactions were accomplished in the sample storage compartment of the PA 800 Plus instrument. The temperature of the compartment was adjusted according to the individual enzyme reaction requirement between 40 °C and 60 °C. An external wireless temperature sensor (±0.5 °C) was utilized to precisely follow the temperature of the sequencing reaction mixtures. Exoglycosidase digestions were performed by the addition of 1.0 µL of sialidase (5.0 mU), 1.0 µL of β-galactosidase (25 mU) and 1.0 µL of β-N-Acetyl-hexosaminidase (25 mU) to 50 µL of glycan sample containing reaction mixture (released from 100 µg glycoprotein), consecutively (full-automation) of in a premixed array (semi-automation).
